# Microstructural Evolution of Pearlitic Wheel Steel Under Thermal–Mechanical Fatigue

**DOI:** 10.3390/ma19132881

**Published:** 2026-07-06

**Authors:** Mingzhe Fan, Yuming Fu, Guang Li, Xiang Li, Sa Zhao, Zhifeng Li, Guanzhen Zhang, Chi Zhang

**Affiliations:** 1 Metals and Chemistry Research Institute, China Academy of Railway Sciences Corporation Limited, Beijing 100081, China; mingzhefan_sjtu@163.com (M.F.); 19062186468@163.com (G.L.); tklixiang@yeah.net (X.L.); 18132790106@163.com (S.Z.); 2 Key Laboratory of Advanced Materials of Ministry of Education, School of Materials Science and Engineering, Tsinghua University, Beijing 100084, China; fuym22@mails.tsinghua.edu.cn (Y.F.); chizhang@mail.tsinghua.edu.cn (C.Z.); 3 China Iron and Steel Research Institute Group Co., Ltd., Beijing 100081, China; lizhifengimust@163.com

**Keywords:** pearlitic wheel steel, thermal–mechanical fatigue, cyclic hardening/softening, cementite, dislocation

## Abstract

Pearlitic wheel steel subjected to thermal–mechanical fatigue (TMF) during braking can undergo catastrophic fracture. This study clarifies the microstructural evolution governing the macroscopic cyclic hardening/softening behavior of pearlitic wheel steel under thermal–mechanical fatigue (TMF) with a constant mechanical strain range of −0.4% to +0.2%. At lower temperature amplitudes (200–500 °C), the geometrically necessary dislocation (GND) density reaches 20.4 × 10^14^/m^2^ during initial cycles, corresponding to cyclic hardening due to dislocation pile-ups at cementite lamellae interfaces. With increasing cycles, the GND density decreases to 12.3 × 10^14^/m^2^, concurrent with softening arising from lamellar bending/fracture, partial spheroidization, and dynamic recrystallization of ferrite. At higher temperature amplitudes (200–730 °C), the GND density decreases from 8.8 × 10^14^/m^2^ to 3.5 × 10^14^/m^2^, reflecting sustained cyclic softening dominated by thermally activated mechanisms, including cementite spheroidization and dislocation annihilation. The resulting softened microstructure consists of ferrite grains, intragranular dispersed cementite, and chain-like coarse cementite at boundaries. Unlike previous studies that focused on single loading conditions (e.g., thermal fatigue, rolling contact fatigue, or wear), the present work addresses the more complex TMF scenario and quantitatively elucidates the interplay between mechanical response and microstructural evolution in pearlitic steel. This work provides theoretical guidance for the development of a fatigue life prediction model for pearlitic wheels under braking.

## 1. Introduction

Pearlitic steel has been widely employed as the primary material for railway wheels due to its excellent combination of strength, toughness, and wear resistance [1,2]. The characteristic lamellar structure, consisting of alternating ferrite and cementite plates, endows the steel with high resistance to rolling contact fatigue and sliding wear, which are essential for safe and durable service under repeated wheel–rail contact and braking conditions. Consequently, pearlitic wheels have become the industry standard in many railway networks worldwide. The service life of railway wheels is critical to the safety of railway systems; therefore, understanding the degradation mechanisms of pearlitic wheel steel is of paramount importance.

During braking operations, railway wheel rims are subjected not only to cyclic mechanical strains but also to repeated thermal cycles, as surface temperatures rise rapidly during braking and cool down afterwards [3]. Under certain extreme braking conditions, the peak temperature can exceed 700 °C [3]. During heating, the restriction of thermal expansion induces circumferential compressive stress in the wheel. In contrast, during the rapid cooling process after braking, the restriction of thermal contraction generates a circumferential tensile stress [4]. Consequently, the wheel tread is subjected to out-of-phase thermal–mechanical fatigue (TMF) loading characterized by compression at elevated temperatures and tension at lower temperatures during braking. Compared to conventional fatigue [5,6,7], the interplay between cyclic plasticity and transient thermal fields gives rise to more complex damage processes that cannot be adequately captured by studying thermal exposure or mechanical cycling alone. Therefore, understanding TMF behavior is essential for reliable life prediction of pearlitic wheels under service-relevant braking conditions.

The microstructural evolution and mechanical response of pearlitic steel have been extensively investigated under single-loading conditions, including isothermal fatigue [8], rolling-contact fatigue [9,10], and sliding wear [11]. The effects of thermal exposure alone on cementite spheroidization and ferrite recovery have also been well documented [12,13]. However, studies on the TMF of pearlitic steel, where cyclic temperature variations are superimposed on mechanical straining, remain scarce. In contrast, TMF behavior has been systematically explored for other material systems, such as nickel-based single-crystal superalloys [14,15] and martensitic steels [16,17], where the coupled effects of temperature cycling and mechanical loading on damage mechanisms have been extensively characterized. The corresponding TMF response of pearlitic steel has received limited attention. Therefore, there is an urgent need for experimental research dedicated to understanding the coupled degradation mechanisms of pearlitic wheel steel under TMF conditions.

This study systematically investigates the correlation between the mechanical response and microstructural evolution of pearlitic wheel steel under simulated braking cycles through half-life and full-life strain-controlled TMF tests. This work focuses on the cyclic hardening/softening behavior, linking each stage of the mechanical response to specific microstructural evolution. Unlike previous studies that primarily examined pearlitic steel under single-loading conditions or focused TMF investigations on other material systems, this work uniquely quantifies the microstructural evolution mechanisms of pearlitic steel under TMF. This work provides theoretical guidance on the damage mechanisms, allowing us to establish a fatigue life prediction model of pearlitic wheels under braking conditions.

## 2. Materials and Methods

**Figure 1:**
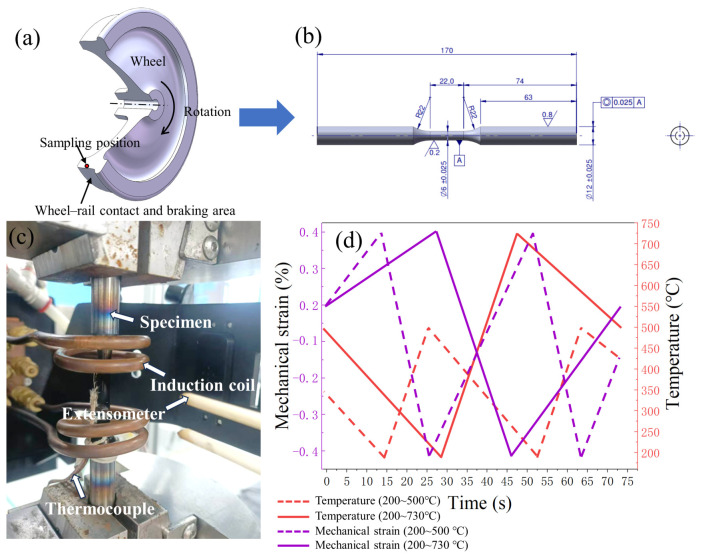
(**a**) A schematic diagram of the sampling points for the TMF test specimens; (**b**) the dimension of the TMF specimens; (**c**) the TMF testing system used in this work; (**d**) the loading path of TMF tests.

This study utilized industrially produced railway wheels, and their chemical composition is detailed in Table 1. The position and dimensions of the TMF specimens within the wheel are shown in Figure 1a,b, respectively. The TMF specimens were extracted from the near-tread region of the railway wheel. The TMF tests were strictly conducted in accordance with ASTM E2368-10 (2017) [18]. Testing was performed on a servo-hydraulic testing machine (MTS Systems Corporation, Eden Prairie, MN, USA) equipped with hydraulic grips (Instron, Norwood, MA, USA) and an induction heating system (Servotest, Egham, UK). Temperature control and measurement were achieved using K-type thermocouples spot-welded onto the specimens (Omega Engineering, Stamford, CT, USA), while axial strain was measured by using a water-cooled high-temperature extensometer (Epsilon Technology Corp., Jackson, WY, USA). The TMF testing system is shown in Figure 1c. All tests were carried out under fully reversed phase conditions, where the maximum mechanical strain coincided with the minimum temperature. Two distinct temperature cycles were applied: (1) temperature varying between 200 °C and 500 °C, and (2) temperature varying between 200 °C and 730 °C. A constant mechanical strain range of −0.4% to +0.2% was maintained for all tests. The mechanical strain range of −0.4% to +0.2% was selected to simulate the combined tensile and compressive hoop strains experienced by the wheel tread during a typical braking cycle. The two temperature amplitudes, 200–500 °C and 200–730 °C, represent moderate and extreme braking conditions, respectively. The lower range corresponds to routine braking where the peak temperature is lower, while the higher range covers emergency or downhill braking where the tread surface can exceed 700 °C, leading to rapid microstructural degradation.

Figure 1d shows the load path of TMF tests. Each temperature amplitude underwent both half-life and full-life tests. The cycle frequency was 0.025 Hz for 200–500 °C and 0.014 Hz for 200–730 °C using triangular waveforms for both temperature and mechanical strain, ensuring the same heating and cooling rates (30 °C/s). No dwell times were applied. The out-of-phase condition was maintained with the temperature maximum coinciding with the compressive mechanical strain peak. Each group of experiments was duplicated to eliminate experimental errors (at least three times). To ensure precise control of the mechanical strain, the free thermal expansion behavior for each temperature cycle was first determined through a stress-free test, and this was compensated for in real time during the subsequent TMF tests.

For the lower temperature amplitude (200–500 °C), the average fatigue life ($N_{f}$) was 1121 cycles. Microstructural characterization was performed after 600 cycles (approximately 50% of $N_{f}$) and after full-life failure (1121 cycles). For the higher temperature amplitude (200–730 °C), the average fatigue life was 2762 cycles. Characterization was conducted after 1300 cycles (approximately 50% of $N_{f}$) and after full-life failure (2762 cycles).

## 3. Results and Discussion

### 3.1. Initial Microstructure

**Figure 2:**
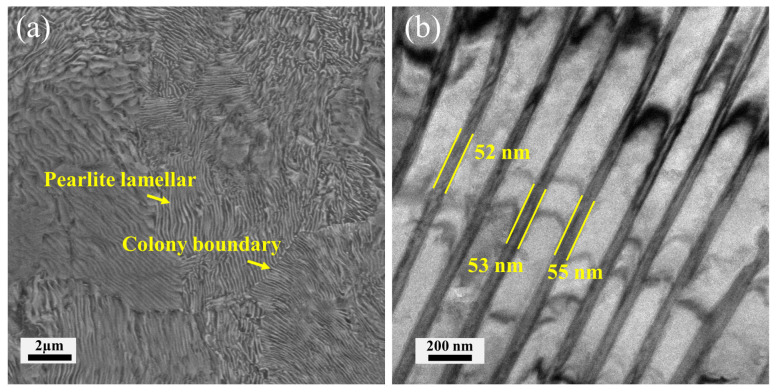
Microstructure of as-received wheel steel. (**a**) SEM image (SE mode); (**b**) TEM bright-field image.

The initial microstructure of the as-received wheel steel is shown in Figure 2. The SEM image reveals multiple pearlite colonies with distinct lamellar orientations, as shown in Figure 2a. Within a single colony, the TEM image resolves the characteristic lamellar architecture consisting of alternating ferrite (α-Fe) and cementite (Fe_3_C) plates, as shown in Figure 2b. The average thickness of cementite is measured to be approximately 53 ± 2 nm (average of 20 measurements). No evidence of spheroidization or significant dislocation activity is observed in the as-received condition, indicating a well-preserved lamellar morphology prior to TMF testing.

### 3.2. Mechanical Response

**Figure 3:**
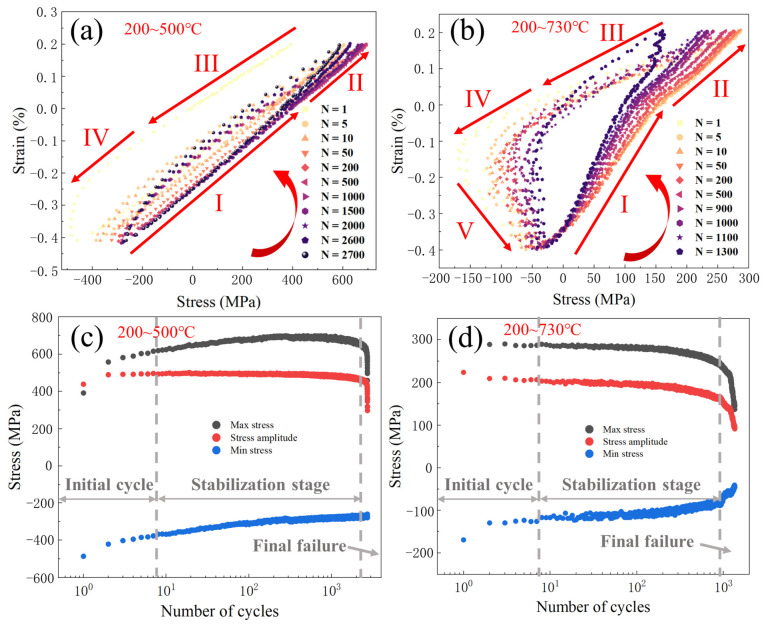
Mechanical responses of TMF tests. Strain–stress curves of TMF tests under different numbers of cycles of (**a**) 200–500 °C and (**b**) 200–730 °C; max stress, stress amplitude and min stress vs. number of cycles at (**c**) 200–500 °C and (**d**) 200–730 °C.

The mechanical response results obtained under the same strain amplitude (−0.4% to +0.2%) but different temperature amplitudes (200–500 °C and 200–730 °C) are shown in Figure 3. Figure 3a,b present the evolution of strain and stress under thermal cycles with an identical base temperature (200 °C) and peak temperatures of 500 °C and 730 °C, respectively. In Figure 3a, the hysteresis loop for a single cycle is divided into four distinct stages (I–VI). During Stage I, the specimen is unloaded from the maximum compressive strain and subjected to initial tensile loading. This stage involves elastic responses during unloading and the beginning of tensile loading. The inverse of the slope of the fitted curve in this region represents the elastic modulus. When the tensile strain reaches a level sufficient to induce plasticity under the given temperature and strain rate, plastic deformation commences (Stage II). The apparent elastic modulus decreases, analogous to conventional material hardening behavior. In Stage III, the specimen is unloaded from the maximum tensile strain and then exhibits an elastic response during compressive loading, accompanied by a gradual temperature increase. Upon the compressive strain reaching the threshold for plasticity under the prevailing conditions, the specimen enters Stage IV, where plastic deformation occurs. The compressive stress at which plasticity initiates is significantly lower than the tensile stress due to the higher temperature. A reduction in the apparent elastic modulus is also observed in Stage IV.

The hysteresis loop observed at a peak temperature of 730 °C differs significantly from that at 500 °C (Figure 3b). At the higher peak temperature, plastic deformation in Stage II is more pronounced, indicating a higher degree of microstructural softening induced by temperature. Furthermore, as the temperature continues to rise during Stage IV, thermal activation softening dominates material deformation, leading to the transition into Stage V. In this stage, the stress gradually decreases with increasing strain. With accumulating fatigue cycles, the shape and size of the hysteresis loop undergo significant changes. Overall, the loop narrows and its area diminishes, indicating a reduction in energy dissipation per cycle and confirming the occurrence of cyclic softening in the material. Specifically, the elastic modulus in Stage I decreases with increasing cycle number, and the peak tensile and compressive stresses also exhibit a declining trend throughout the cycling process. As the number of cycles increases, the area of the hysteresis loop shows significant change.

To better investigate the cyclic hardening/softening behavior of the material under thermo-mechanical fatigue, the variations in peak tensile stress (max stress), peak compressive stress (min stress), and stress amplitude with increasing fatigue cycles are plotted in Figure 3c,d. For the specimen tested at a peak temperature of 500 °C, the peak tensile stress initially increased and then decreased with an increasing number of cycles, while the peak compressive stress exhibited a continuous decrease. This indicates that during the low-temperature tensile phase, strain hardening dominated the deformation in the initial cycling stage, but softening mechanisms became predominant in the mid-to-later stages. In contrast, during the high-temperature compressive phase, the relatively elevated temperature led to sustained softening. Under the synergistic effect of cyclic hardening and softening, the stress amplitude first increased rapidly, then entered a nearly stable stage, and finally decreased rapidly. This evolution suggests that the microstructure initially underwent hardening, followed by a quasi-dynamic balance between hardening and softening mechanisms. Due to the relatively low temperature amplitude, dislocation multiplication and pile-up dominated the material’s response in the initial stage (N < 1% $N_{f}$, where $N_{f}$ is defined as the fatigue life). During the stabilization stage (N < 90% $N_{f}$), a dynamic balance was established between softening and hardening. In the final stage of cycling (N **>  90% $N_{f}$), the specimens entered rapid fracture.

For the specimen tested at a peak temperature of 730 °C, the peak tensile stress, peak compressive stress, and stress amplitude all decreased with increasing fatigue cycles, indicating that microstructural softening dominated throughout the entire process. In the initial stage of cycling (N  < 1% $N_{f}$), a sharp decline in stress was observed, suggesting significant cyclic softening. As cycling progressed into the stabilization stage (N* * <  90% $N_{f}$), the stress decreased gradually. This phase represents a quasi-stable yet continuously degrading state, occupying the majority of the fatigue life. The slow stress reduction in this stage may be attributed to softening mechanisms with slower kinetics, such as carbide coarsening and dynamic recrystallization of ferrite. In the final failure stage (N* * >  90% $N_{f}$), the rate of stress reduction accelerated noticeably. This accelerated decline likely marks the accumulation of macroscopic damage, such as the initiation of micro-cracks and micro-voids, which progressively reduced the material’s load-bearing capacity. The final failure stage is characterized by a rapid, sharp drop in stress until fracture occurred, which is a typical manifestation of unstable propagation of macroscopic cracks.

### 3.3. Microstructural Evolution

#### 3.3.1. Temperature Amplitude Under 200–500 °C

**Figure 4:**
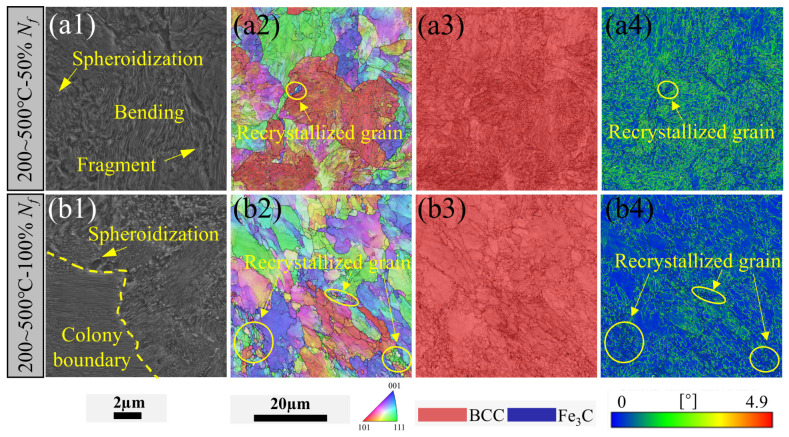
SEM and EBSD results of pearlitic wheel steel under 200–500 °C. (**a1**–**a4**) SEM image, inverse pole figure (IPF) map, phase map and KAM map of half-life (50% $N_{f}$) specimen; (**b1**–**b4**) SEM image, IPF map, phase map and KAM map of full-life (100% $N_{f}$) specimen.

Figure 4 presents the SEM and EBSD characterization results of specimens subjected to TMF under different temperature ranges, showing the microstructural features at half-life and full-life stages. At a temperature amplitude of 200–500 °C, Figure 4(a1,a2) reveal that carbon preferentially diffuses to colony boundaries, where initial spheroidization occurs, whereas the lamellar structure is largely retained within the colonies. The temperature-driven spheroidization process of cementite can be explained by thermodynamics [12]. The spheroidization process is governed by capillarity-driven volume diffusion. The thermodynamic driving force originates from local curvature differences: carbon atoms have a higher chemical potential at sharp edges and corners (high curvature) and a lower potential on flat surfaces (low curvature). Consequently, spheroidization initiates preferentially at the edges and corners, where carbon diffuses away, causing edge recession and the formation of thick ridges. Subsequently, a reverse diffusion flux develops between the flat regions and the ridges, leading to local thinning and the nucleation of holes in the flat surfaces. These holes grow and coalesce, eventually fragmenting the plate into multiple spheroids [12]. In contrast to the extensive shear fracture of cementite lamellae observed under room-temperature deformation, bending of the lamellae is evident in the 200–500 °C range, indicating coordinated deformation with the ferrite matrix. Moreover, only limited lamellar fracture and carbon dissolution take place. The soft ferrite phase accommodates most of the strain, leading to substantial dislocation multiplication [19]. Meanwhile, the lamellar cementite strongly impedes dislocation glide, causing dislocation pile-ups at ferrite/cementite interfaces. The relationship between the evolution process of cementite and the mechanical behavior is discussed below.

Intact cementite lamellae act as effective barriers to dislocation glide, forcing Orowan-type looping and dislocation pile-ups that generate substantial strengthening [20]. Upon lamellar bending and fracture, this barrier function is lost. Dislocations can bypass fragmented cementite particles more easily, and the mean free path for dislocation motion increases, directly reducing the dislocation-induced back stress contribution to flow stress. Moreover, as cementite spheroidization proceeds, the lamellar interface area decreases dramatically. Incoherent ferrite/cementite interfaces are less effective at impeding dislocation motion than the original lamellar interfaces, contributing further to softening.

The kernel average misorientation (KAM) map (Figure 4(a4)) reveals significant dislocation activity and an increased lattice orientation mismatch. Concurrently, a small number of distortion-free recrystallized grains are observed. Distortion-free ferrite grains cannot sustain the same level of dislocation-induced back stress as the deformed matrix, and they deform more easily under subsequent cycling, reducing the macroscopic flow stress.

With an increasing number of cycles, extensive spheroidization proceeds at colony boundaries (Figure 4(b1)). Concurrently, cementite lamellae within the pearlite colonies become uniformly thin and fracture. As shown in Figure 4(b2,b4), the ferrite phase undergoes lattice rotation and elongation, while the intragranular dislocation density decreases significantly. Dislocation pile-ups promote substantial carbon dissolution, forming supersaturated ferrite and causing the disappearance of the lamellar structure. The accumulated cyclic strain energy, together with the elevated temperature, provides the driving force for dynamic recrystallization. The dislocation cell structures within ferrite grains progressively transform into distortion-free dynamically recrystallized grains. Softening mechanisms, including dislocation annihilation, continuous dynamic recrystallization, and cementite dissolution, become dominant, which is macroscopically manifested as gradual cyclic softening. It is worth noting that the EBSD technique is unable to resolve the lamellar carbides (Figure 4(a3,b3)).

**Figure 5:**
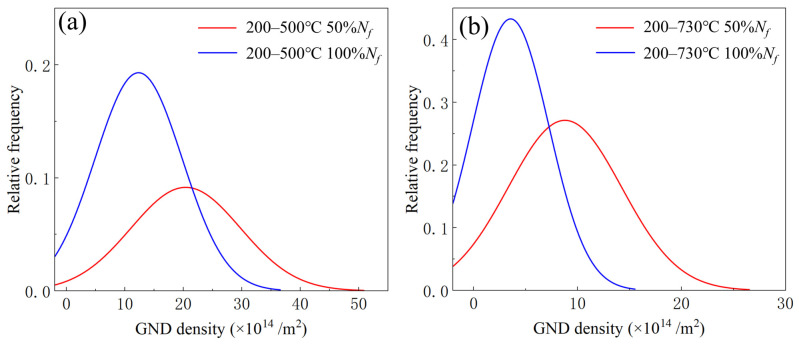
The frequency distribution of GND density under (**a**) 200–500 °C and (**b**) 200–730 °C.

The GND density ρ_GND_ can provide a direct measure of material hardening/softening [21]. It can be derived from kernel average misorientation (KAM) maps via the misorientation angle with the following equation [22]:

ρ_GND_ = αθ/bx (1)

where b is the magnitude of the Burgers vector for dislocations in ferrite. For α-Fe, b = 0.248 nm. x is the step size of the EBSD scan. A step size of 75 nm was used for all KAM maps in this study. θ is the local misorientation angle obtained from the KAM map. α is a dimensionless constant related to the dislocation arrangement and the strain gradient geometry. Based on the strain gradient plasticity theory and literature practice for pearlitic steels, we set α = 3 [23]. As shown in Figure 5a, at 200–500 °C, the GND density is as high as 20.4 × 10^14^/m^2^ during the initial cycles and drops to 12.3 × 10^14^/m^2^ with increasing cycles. The flow stress contribution from dislocations scales with the square root of the total dislocation density [24]. The initially high GND density of 20.4 × 10^14^ m^−2^ arises from GND generated to accommodate plastic strain gradients near ferrite/cementite interfaces, leading to an elevated dislocation-induced back stress that strengthens the ferrite matrix [25]. This GND-driven strengthening manifests macroscopically as the observed initial cyclic hardening (Figure 3c). As cycling proceeds, thermally assisted recovery processes, including dislocation climb, cross-slip and annihilation, gradually reduce the GND density to 12.3 × 10^14^ m^−2^ [26]. This decrease lowers the dislocation storage capacity and reduces the athermal component of flow stress, allowing softening mechanisms to dominate [27]. Thus, the quantitative GND density evolution can directly capture the transition from dislocation multiplication-dominated hardening to recovery-dominated softening. It is worth noting that the KAM-based GND density is a semi-quantitative measure. Its accuracy is influenced by EBSD step size, the misorientation threshold, and the choice of α. KAM does not separate GNDs from statistically stored dislocations (SSDs) and may underestimate density in fine substructures [28]. However, with consistent parameters, it provides reliable relative trends for dislocation activity.

**Figure 6:**
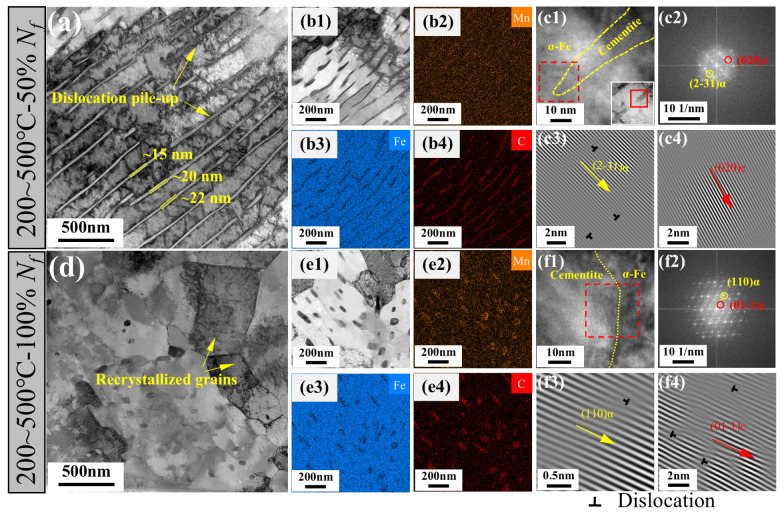
TEM results of pearlite wheel steel under 200–500 °C. Half-life (50% $N_{f}$) specimen: (**a**) TEM bright-field image; (**b1**–**b4**) EDS maps; (**c1**) HRTEM image of fractured cementite edge, (**c2**) FFT image of red-dashed box in (**c1**), and (**c3**,**c4**) IFFT image of (2-31)α and (020)c vectors, respectively. Full-life (100% $N_{f}$) specimen: (**d**) TEM bright-field image; (**e1**–**e4**) EDS maps; (**f1**) HRTEM image of cementite edge, (**f2**) FFT image of red-dashed box in (**f1**), and (**f3**,**f4**) IFFT image of (110)α and (01-1)c vectors, respectively.

To further characterize the microstructural evolution during TMF, FIB was used to prepare thin specimens for subsequent TEM analysis. Figure 6a shows that at 200–500 °C with 50% $N_{f}$, the lamellar structure is largely retained. Partial fracture and thinning (from 52–55 nm to 15–22 nm) of cementite lamellae are observed, accompanied by significant dislocation pile-ups at ferrite/cementite interfaces. TEM combined with energy-dispersive X-ray spectroscopy (EDS) mapping of the lamellar structure (Figure 6(b1–b4)) confirms enrichment of C and Mn in cementite, consistent with the typical elemental partitioning behavior between ferrite and cementite in pearlitic steels. Notably, dislocation lines coincide with elemental segregation, particularly of C. Although direct experimental evidence for pipe diffusion under the present TMF conditions is lacking, this spatial correlation is consistent with the well-documented mechanism whereby carbon atoms are preferentially absorbed by dislocation cores due to their lower activation energy for diffusion [29]. Specifically, Liu et al. [29] reported that in pearlitic steels subjected to dynamic loading, carbon atoms at ferrite/cementite interfaces are dragged out by dislocation loops, leading to cementite decomposition. Thus, the observed dislocation–carbon coincidence suggests that pipe diffusion along dislocations may facilitate lamellar fracture and dissolution.

High-resolution TEM (HRTEM) analysis was performed on a fractured cementite edge (Figure 6(c1–c4)). The fast Fourier transform (FFT) pattern obtained from the red-dashed region in Figure 6(c1) is shown in Figure 6(c2), where diffraction vectors (2-31)α for α-Fe and (020)c for cementite are identified. Subsequent inverse FFT (IFFT) processing (Figure 6(c3,c4)) reveals dislocations within the α-Fe phase, supporting the solute-drag model as a viable mechanism for lamellar fragmentation under thermo-mechanical cycling [29]. With increasing cycles, progressive spheroidization of cementite and the formation of recrystallized grains are observed (Figure 6d), in agreement with the SEM and EBSD results. EDS maps (Figure 6(e1–e4)) show C and Mn segregation predominantly in cementite, while the dislocation density in the matrix significantly decreases, consistent with KAM analysis. HRTEM of a cementite terminus (Figure 6(f1)) and its FFT pattern (Figure 6(f2)) identifies the (110)α and (01-1)c diffraction vectors. IFFT images (Figure 6(f3,f4)) unexpectedly reveal dislocations within the cementite phase itself. This suggests that under thermal activation, slip systems in cementite may be activated, contributing to C diffusion and decomposition not only at interfaces but also within the cementite interior, potentially via dislocation-assisted carbon transport [29]. Although the density of such dislocations appears limited, their presence is notable because cementite is generally considered brittle and resistant to dislocation glide at low temperatures. The thermally activated slip within cementite may provide an additional pathway for carbon migration, accelerating lamellar fragmentation and spheroidization beyond what interface-only diffusion would predict. Nevertheless, direct evidence for this mechanism remains tentative, and further work is required to confirm whether these dislocations are truly mobile and contribute to carbon transport under cyclic thermal–mechanical loading.

In summary, from the perspective of microstructural evolution, the macroscopic mechanical behavior is determined by the synergistic effect of hardening and softening mechanisms. During the initial cycles, the GND density increases rapidly while cementite spheroidization and dynamic recrystallization remain insufficient, leading to macroscopic cyclic hardening. With a further increase in the number of cycles, the GND density decreases. Cementite spheroidization and dynamic recrystallization intensifies, resulting in macroscopic cyclic softening.

#### 3.3.2. Temperature Amplitude Under 200–730 °C

**Figure 7:**
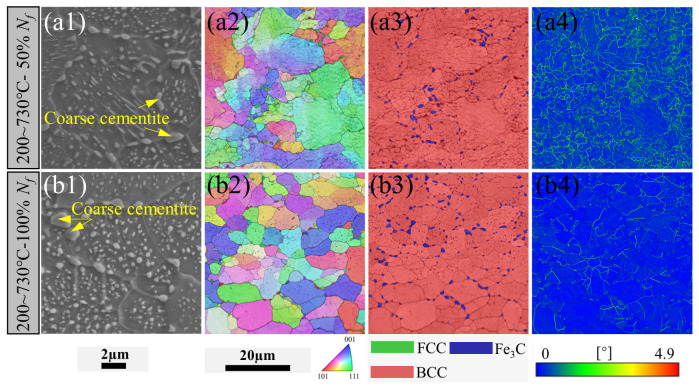
SEM and EBSD results of pearlite wheel steel under 200–730 °C. (**a1**–**a4**) SEM image, inverse pole figure (IPF) map, phase map and KAM map of half-life (50% $N_{f}$) specimen; (**b1**–**b4**) SEM image, IPF map, phase map and KAM map of full-life (100% $N_{f}$) specimen.

At the higher temperature amplitude (200–730 °C), the GND density decreased from 8.8 × 10^14^/m^2^ to 3.5 × 10^14^/m^2^ with increasing cycles (Figure 5b), indicating that the dynamic recovery rate exceeded the dislocation multiplication rate and sustained cyclic softening. Consequently, the ferrite could not accumulate the critical strain necessary for conventional recrystallization. KAM maps (Figure 7(a4)) indicate a relatively low dislocation density. The elevated temperature significantly enhanced carbon diffusion rates, accelerating both the spheroidization and coarsening of cementite [12]. Dislocations tended to pile up at high-angle boundaries during the low-temperature phase of the cycle. This provided fast diffusion paths for carbon, leading to the formation of large (0.6–1.1 μm) chain-like spheroidized cementite particles at these boundaries [30], which were detectable by SEM and EBSD (Figure 7(a1–a3)). As shown in Figure 7(a3), partial spheroidization of cementite occurred within the pearlite colonies, while some regions retained their lamellar morphology. With an increasing number of cycles, the fraction and size of coarse cementite increased, and the cementite within the original pearlite colonies spheroidized completely (Figure 7(b1–b3)). Compared to the 200–500 °C specimen, the fatigue process at 200–730 °C was dominated by softening mechanisms such as cementite spheroidization/coarsening and dislocation annihilation (Figure 7(b4)). The final microstructure consisted of ferrite grains, intra-granular finely dispersed spheroidized cementite, and chain-like coarse spheroidized cementite at grain boundaries. Macroscopically, the ongoing coarsening of grain boundary cementite and spheroidization within the pearlite colonies led to continuous cyclic softening.

**Figure 8:**
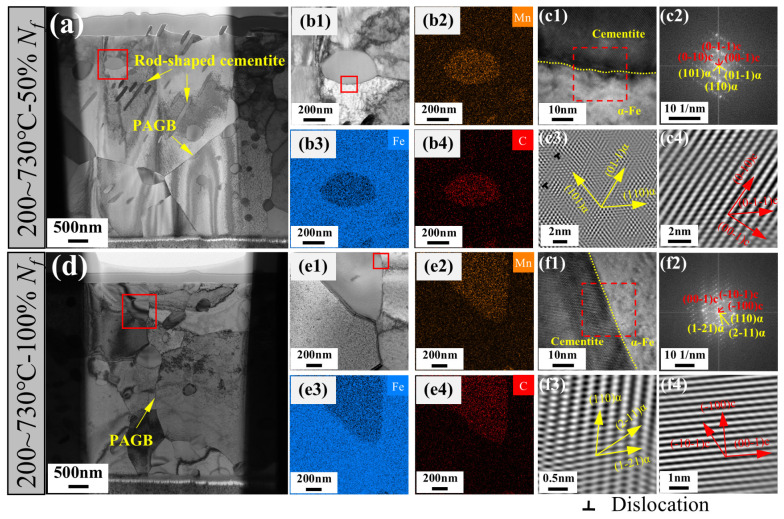
TEM results of pearlite wheel steel under 200–730 °C. Half-life (50% $N_{f}$) specimen: (**a**) TEM bright-field image; (**b1**–**b4**) EDS maps of coarse cementite; (**c1**) HRTEM image of the red box in (**b1**), (**c2**) FFT image of red-dashed box in (**c1**), and (**c3**,**c4**) IFFT image of (**c2**). Full-life (100% $N_{f}$) specimen: (**d**) TEM bright-field image; (**e1**–**e4**) EDS maps of coarse cementite; (**f1**) HRTEM image of the red box in (**e1**), (**f2**) FFT image of red-dashed box in (**f1**), and (**f3**,**f4**) IFFT image of (**f2**). PAGB: Prior Austenite Grain Boundary.

Figure 8a shows a TEM bright-field image at 200–730 °C and 50% $N_{f}$. Consistent with SEM and EBSD observations, cementite has evolved into rod-like and spheroidized morphologies with larger dimensions than those observed at lower temperature amplitudes. EDS mappings of a coarse cementite particle (red box in Figure 8a, shown in Figure 8(b1–b4)) and HRTEM of its boundary (Figure 8(c1–c4)) reveal no dislocations within the cementite, indicating that dissolution and spheroidization in this case are primarily driven by thermal activation. With increasing cycles, cementite at grain boundaries continues to coarsen, and rod-shaped cementite diminishes. EDS analysis of another coarse cementite (red box in Figure 8d, shown in Figure 8(e1–e4)) confirms C and Mn enrichment. HRTEM (Figure 8(f1)) and the corresponding FFT pattern (Figure 8(f2)) from the red-dashed region show distinct diffraction spots for α-Fe and cementite. IFFT images (Figure 8(f3,f4)) reveal well-ordered atomic arrangements in both phases with minimal dislocations, further confirming that under conditions of high temperature amplitude, microstructural evolution is dominated by thermally activated softening mechanisms.

Based on the above evidence, the relationship between the mechanical response and microstructural evolution under TMF can be summarized as follows. At lower temperature amplitudes (200–500 °C), substantial dislocation proliferation during the initial cycles leads to cyclic hardening. As the number of cycles increases, extensive fracture and spheroidization of cementite occur, resulting in cyclic softening. At higher temperature amplitudes (200–730 °C), thermally activated diffusion dominates cementite spheroidization with significantly reduced dislocation activity, leading to sustained cyclic softening. Thus, the competition between dislocation-mediated and thermally activated mechanisms, dictated by the temperature amplitude, directly governs the macroscopic mechanical response and fatigue life of pearlitic wheels under braking-induced thermo-mechanical fatigue.

The present study provides essential microstructural and mechanistic inputs that can improve the physical basis of energy-based life prediction models for TMF. In particular, our quantitative characterization of GND density evolution, cementite lamellar fracture/spheroidization, and ferrite dynamic recrystallization under different temperature amplitudes reveals how the stored strain energy is partitioned between dislocation-mediated hardening and thermally activated softening. These findings can be used to refine the calculation of the total strain energy (e.g., the perfect hysteresis energy and the shape factor) in existing models, such as the model validated for single-crystal superalloys [14]. By linking specific microstructural parameters to the macroscopic cyclic stress–strain response, our work offers a material-oriented foundation for normalizing TMF data across different loading conditions, thereby contributing to the development of more accurate and universally applicable fatigue life prediction methods for pearlitic wheel steels under braking-induced TMF.

## 4. Conclusions

In this study, the microstructural evolution during thermal–mechanical fatigue (TMF) of pearlitic steel was investigated. A particular focus was placed on the relationship between mechanical response and microstructural evolution. This work can provide theoretical guidance on the damage mechanisms, enabling the establishment of a fatigue life prediction model of pearlitic wheels under braking conditions. The principal conclusions are as follows:

(1) At the lower temperature amplitude (200–500 °C) during TMF, the pearlitic steel exhibits an initial cyclic hardening phase followed by gradual softening. The initial hardening arises from dislocation multiplication in the ferrite and its pile-up at cementite lamellae interfaces. The subsequent softening is associated with the bending, fracture, and partial spheroidization of cementite lamellae, as well as continuous dynamic recrystallization of the ferrite grains.
(2) At the higher temperature amplitude (200–730 °C), the pearlitic steel exhibits continuous macroscopic cyclic softening, fundamentally driven by thermally activated microstructural evolution. With increasing cycles, cementite undergoes rapid spheroidization and significant coarsening at grain boundaries, accompanied by dislocation annihilation. These processes collectively lead to a softened microstructure consisting of ferrite grains, intra-granular dispersed cementite, and chain-like coarse cementite at boundaries, resulting in progressive degradation of macroscopic load-bearing capacity.

For braking cycles that cause limited temperature rise (e.g., routine braking), design strategies of wheel materials should consider delaying softening to schedule maintenance or apply surface treatments that delay cementite fragmentation. Under severe braking (peak temperatures > 700 °C, e.g., emergency stops or downhill braking), the wheel rim softens continuously. Therefore, for wheels operating on routes with frequent severe braking, alloys with higher cementite stability should be considered. Additionally, the observed relationship between GND density drop and softening can serve as a quantitative indicator for assessing microstructural degradation in non-destructive inspection protocols.

## Figures and Tables

**Table 1:** Chemical composition of experimental steel (wt.%).

C	Mn	Si	Ni	Mo	V	Cr	Fe
0.67	1.20	0.15	0.25	0.10	0.04	0.50	Balance

## Data Availability

The original contributions presented in this study are included in the article. Further inquiries can be directed to the corresponding author.

## References

[1] Miranda R.S., Agudelo J.I.P., Rezende A.B., Carvalho A.C., Sinatora A., Mei P.R. (2025). Microstructural and Mechanical Characterization of the Tribologically Transformed Zone of Pearlitic and Bainitic Railroad Wheel Steels: Analysis of WEL, TL and NS. Wear.

[2] Esmaeili A., Ahlström J., Ekh M., Nikas D., Vernersson T. (2021). Modelling of Temperature and Strain Rate Dependent Behaviour of Pearlitic Steel in Block Braked Railway Wheels. Railw. Eng. Sci..

[3] Zhang J., Zuo J. (2025). Numerical Thermo-Mechanical Modeling of Tread Braking Systems under Ultra-Long Downhill Constant-Speed Conditions with Coupled Time-Dependent Heat Partition Coefficients. Int. Commun. Heat Mass Transf..

[4] Steyn E., Ahlström J. (2024). Thermo-Mechanical Response of near-Pearlitic Steel Heated under Restriction of Thermal Expansion. J. Mater. Res. Technol..

[5] Hutchinson C.R., De Geuser F., Chen Y., Deschamps A. (2014). Quantitative Measurements of Dynamic Precipitation during Fatigue of an Al–Zn–Mg–(Cu) Alloy Using Small-Angle X-Ray Scattering. Acta Mater..

[6] Shah S., Thronsen E., Hatzoglou C., Wenner S., Marioara C.D., Holmestad R., Holmedal B. (2022). Effect of Cyclic Ageing on the Early-Stage Clustering in Al–Zn–Mg(-Cu) Alloys. Mater. Sci. Eng. A.

[7] Shah S., Gopal A., Thronsen E., Hatzoglou C., Holmedal B. (2022). Precipitation, Mechanical Properties and Early Slant Ductile Fracture in Cyclic and Naturally Aged Al-Zn-Mg(-Cu) Alloys. Mater. Des..

[8] Sistaninia M., Maierhofer J., Spalek A., Gänser H.-P., Bucher C., Pippan R., Mayer H., Schönbauer B.M. (2024). Influence of Surface Condition, Cycling Frequency and Ferritic Zones on the High and Very High Cycle Fatigue Properties of a Pearlitic Steel. Mater. Sci. Eng. A.

[9] Masoumi M., Sinatora A., Goldenstein H. (2019). Role of Microstructure and Crystallographic Orientation in Fatigue Crack Failure Analysis of a Heavy Haul Railway Rail. Eng. Fail. Anal..

[10] Kumar S., Paul S.K., Tiwari M. (2025). Rolling-Sliding Contact Fatigue Behaviour of Pearlitic Steel under a Higher Slide-to-Roll Ratio While the Traction Process. Prog. Eng. Sci..

[11] Hu Y., Zhou L., Ding H.H., Lewis R., Liu Q.Y., Guo J., Wang W.J. (2021). Microstructure Evolution of Railway Pearlitic Wheel Steels under Rolling-Sliding Contact Loading. Tribol. Int..

[12] Amos P.G.K., Bhattacharya A., Nestler B., Ankit K. (2018). Mechanisms of Pearlite Spheroidization: Insights from 3D Phase-Field Simulations. Acta Mater..

[13] Russo M., Saulot A., Sauvage X., Véron M., Rauch E., Thiercelin L., Lebon F., Chantrenne P., Cazottes S. (2025). Multiscale Microstructural Investigations of White and Brown Etching Layers Initiating the Squat Formation in Pearlitic Rail Steels. Mater. Charact..

[14] Tan Z., Zou C., Wang X., Pang J., Li Y., Liu J., Liu J., Li J., Zhang Z., Sun X. (2026). Low-Cycle and Thermal–Mechanical Fatigue of the Fourth-Generation Single Crystal Superalloy: Correlation of Deformation Mechanisms and Life Prediction Method. Int. J. Fatigue.

[15] Segersäll M., Kontis P., Pedrazzini S., Bagot P.A.J., Moody M.P., Moverare J.J., Reed R.C. (2015). Thermal–Mechanical Fatigue Behaviour of a New Single Crystal Superalloy: Effects of Si and Re Alloying. Acta Mater..

[16] Chang L., Li X., Wen J.-B., Zhou B.-B., He X.-H., Zhang G.-D., Xue F., Zhao Y.-F., Zhou C.-Y. (2020). Thermal-Mechanical Fatigue Behaviour and Life Prediction of P92 Steel, Including Average Temperature and Dwell Effects. J. Mater. Res. Technol..

[17] Wen J., Zhou C.-Y., Li X., Pan X.-M., Chang L., Zhang G.-D., Xue F., Zhao Y.-F. (2019). Effect of Temperature Range on Thermal-Mechanical Fatigue Properties of P92 Steel and Fatigue Life Prediction with a New Cyclic Softening Model. Int. J. Fatigue.

[18] (2017). Practice for Strain Controlled Thermomechanical Fatigue Testing.

[19] Zhang X., Godfrey A., Hansen N., Huang X., Liu W., Liu Q. (2010). Evolution of Cementite Morphology in Pearlitic Steel Wire during Wet Wire Drawing. Mater. Charact..

[20] Páez J.R., Dorronsoro A., Martínez-Esnaola J.M., Gil Sevillano J., Alkorta J. (2024). A Microstructure-Based Constitutive Model for Eutectoid Steels. Acta Mater..

[21] Mi B., Yang Z., Chen H., Zhang C. (2023). Quasi In-Situ Observations of Microstructure Evolution during Low Cycle Fatigue in Carbide-Free Bainitic Steel. Int. J. Fatigue.

[22] Moussa C., Bernacki M., Besnard R., Bozzolo N. (2015). About Quantitative EBSD Analysis of Deformation and Recovery Substructures in Pure Tantalum. IOP Conf. Ser. Mater. Sci. Eng..

[23] Li Y., Goto S., Kostka A., Herbig M. (2023). Local Measurement of Geometrically Necessary Dislocation Densities and Their Strengthening Effect in Ultra-High Deformed Pearlite. Mater. Charact..

[24] Mecking H., Kocks U.F. (1981). Kinetics of Flow and Strain-Hardening. Acta Metall..

[25] Zhang X., Zhao J., Kang G., Zaiser M. (2023). Geometrically Necessary Dislocations and Related Kinematic Hardening in Gradient Grained Materials: A Nonlocal Crystal Plasticity Study. Int. J. Plast..

[26] Wang N., Chen Y., Wu G., Zhao Q., Zhang Z., Zhu L., Luo J. (2022). Non-Equivalence Contribution of Geometrically Necessary Dislocation and Statistically Stored Dislocation in Work-Hardened Metals. Mater. Sci. Eng. A.

[27] Scherbring S., Adams B., Mola J. (2024). Impact of Interlamellar Spacing and Non-Pearlitic Features on Mechanical Properties and Cyclic Damage Initiation in near-Eutectoid Pearlitic Steels. Mater. Sci. Eng. A.

[28] Ateba Betanda Y., Helbert A.-L., Brisset F., Mathon M.-H., Waeckerlé T., Baudin T. (2014). Measurement of Stored Energy in Fe–48%Ni Alloys Strongly Cold-Rolled Using Three Approaches: Neutron Diffraction, Dillamore and KAM Approaches. Mater. Sci. Eng. A.

[29] Liu Y., Zhang S., Feng C., Su X., Chen Y., Jing L. (2024). Dynamic Mechanical Behaviors of Pearlitic U71MnG Rail Steel: Deformation Mechanisms and Constitutive Model. Mater. Sci. Eng. A.

[30] Tian Y., Tan Z., Zhang J., Yuan Z., Zhang X., Zhang Z., Zhang M. (2023). Microstructure Stability in Wheel Steel: A Case of Thermal-Accumulated Damage Capacity in Pearlite and Low-Carbon Bainite. Eng. Fail. Anal..

